# Depolymerized Fucoidan Alleviates High-Fat Diet-Induced Obesity in Association with Alterations in Gut Microbiota and Metabolic Profiles

**DOI:** 10.3390/foods15193508

**Published:** 2026-10-01

**Authors:** Xiaona Sun, Lin Bai, Changyu Su, Chunqing Ai, Xiaomeng Ren, Fanhua Kong, Shuang Song

**Affiliations:** 1 School of Smart New Agricultural Industry, Dalian Art College, Dalian 116600, China; xiaonana1023@126.com; 2 SKL of Marine Food Processing & Safety Control, National Engineering Research Center of Seafood, Collaborative Innovation Center of Seafood Deep Processing, National & Local Joint Engineering Laboratory for Marine Bioactive Polysaccharide Development and Application, Liaoning Key Laboratory of Food Nutrition and Health, School of Food Science and Technology, Dalian Polytechnic University, Dalian 116034, China; bailinxju18@163.com (L.B.); suchyu@163.com (C.S.); acqdongying@163.com (C.A.); rxmfighting@163.com (X.R.); kkong0930@163.com (F.K.)

**Keywords:** seaweed polysaccharides, obesity, gut microbiota, metabolites

## Abstract

Obesity is a major global health concern associated with various metabolic disorders. Fucoidan, a seaweed-derived sulfated polysaccharide, has shown potential in alleviating obesity through modulation of gut microbiota. However, comparative effects of native and depolymerized fucoidan (Dfuc) on HFD-induced obesity and gut microbiota dysbiosis remain unclear. In this study, we compared the anti-obesity effects of fucoidan and Dfuc and evaluated their regulatory effects on gut microbiota and microbial metabolites in HFD-fed mice. Results showed that fucoidan and Dfuc reduced body weight gain, fat accumulation and hepatic lipid abnormalities. They also alleviated liver injury and oxidative stress and inflammation in hepatic and colonic tissues, with Dfuc significantly improving parameters such as serum AST, hepatic MDA, SOD activity, and TNF-α expression compared to the HF group. Furthermore, gut microbiota analysis showed that both fucoidan and Dfuc treatments enriched *Alloprevotella* and *Allobaculum*, while fucoidan increased *Akkermansia* and *Blautia*, and Dfuc promoted *Bifidobacterium* and *Romboutsia*. Moreover, metabolite analysis showed that Dfuc significantly increased fecal acetate and propionate concentrations, and induced more pronounced changes in fecal metabolites related to amino acid, carbohydrate, and bile acid metabolism. These findings suggest that Dfuc holds potential as a functional food ingredient for alleviating HFD-induced obesity and gut microbiota dysbiosis.

## 1. Introduction

Obesity has become a major global public health concern and is closely associated with gut microbiota dysbiosis and metabolic disorders [1]. Long-term high-fat diet (HFD) is an important dietary driver of obesity, promoting excessive fat accumulation and impaired lipid homeostasis [2]. In parallel, HFD reshapes the intestinal microbial community, often favoring potentially harmful and lipopolysaccharide-producing bacteria while reducing beneficial taxa, particularly short chain fatty acid (SCFA)-producing bacteria [3]. These microbial shifts may compromise intestinal barrier integrity and promote metabolic endotoxemia and chronic low-grade inflammation, thereby contributing to obesity progression [4]. In addition, microbial-derived metabolites, e.g., SCFAs, amino acid and bile acids, are involved in regulating host energy metabolism, lipid metabolism, inflammatory responses, and intestinal barrier function [5]. Therefore, modulating gut microbiota and its metabolites is considered a promising strategy for mitigating HFD-induced obesity and related metabolic disorders.

Seaweed polysaccharides, as dietary fibers, are generally resistant to complete degradation by host digestive enzymes, can reach the intestine and interact with gut microbiota, thereby influencing microbial composition and metabolite production [6]. *Undaria pinnatifida* polysaccharides improved body composition, lipid abnormalities, and inflammatory responses in HFD-fed mice through the modulation of intestinal microecology [7]. In addition, *Sargassum fusiforme* polysaccharides ameliorated HFD-induced obesity and hyperlipidemia by remodeling gut microbiota, enriching beneficial taxa such as *Akkermansia*, and regulating bile acid-related metabolism [8]. However, the bioactivities of polysaccharides are closely related to their structural characteristics, particularly molecular weight. Oligosaccharides from *Gracilaria lemaneiformis* showed stronger effects than their native polysaccharides in attenuating HFD-induced metabolic disorders by promoting Bacteroidales proliferation [9]. Similarly, depolymerized sea cucumber sulfated polysaccharide showed a stronger inhibitory effect on fat accumulation than its native form [10]. On the contrary, higher-molecular-weight konjac glucomannan [11] and *Pleurotus citrinopileatus* polysaccharides [12] exhibited more pronounced anti-obesity or lipid-lowering effects than their lower-molecular-weight fractions. These findings suggest that the relationship between molecular weight and bioactivities of polysaccharides is not simply linear.

Fucoidan is a fucose-containing sulfated polysaccharide derived from brown seaweed and has been reported to modulate gut microbiota [1]. Our previous in vitro fermentation study indicated that reducing the molecular weight of fucoidan promoted its microbial utilization and enhanced its regulatory effects on microbial composition and metabolites [13]. However, differences between native fucoidan and depolymerized fucoidan (Dfuc) in regulating HFD-induced obesity, metabolic disorders, and gut microbiota dysbiosis remain unclear in vivo. In the present study, the effects of fucoidan and Dfuc were compared in an HFD-induced obese mouse model. Body weight, lipid profiles, and oxidative stress and inflammatory responses in the liver and colon were evaluated. Meanwhile, gut microbiota composition, SCFAs, and fecal metabolites were analyzed to assess the regulatory effects of fucoidan and Dfuc on gut microbiota. This study aimed to provide new insights into the anti-obesity potential of depolymerized fucoidan, supporting its future application as a functional food ingredient.

## 2. Materials and Methods

### 2.1. Materials and Chemicals

*Laminaria japonica* fucoidan was purchased from Rizhao Jiejing (Rizhao, China). Standard monosaccharides were supplied by Sigma-Aldrich (St. Louis, MO, USA). SCFA standards and an internal standard were provided by Aladdin (Shanghai, China). The remaining reagents were obtained from Macklin (Shanghai, China).

### 2.2. Preparation of Depolymerized Fucoidan (Dfuc)

Photocatalytic degradation reaction was referred to our previous reports [14]. Briefly, 2 g TiO_2_ was dispersed in 400 mL 10 mg/mL fucoidan solution. Subsequently, 15 mL 30% H_2_O_2_ was added, followed by light irradiation to initiate reaction. After 5 h, the TiO_2_ particles were removed by centrifugation (10,000× *g*, 15 min). The supernatant was passed through a molecular weight cut-off (MWCO) of 5 kDa ultrafiltration membrane, and then the <5 kDa penetrant further was dialyzed (MWCO 100 Da), and the part in the dialysis bag was collected, named as Dfuc.

### 2.3. Thin Layer Chromatography (TLC) and Molecular Weight (Mw) Distribution

TLC was used to observe the oligosaccharide profiles [15]. Briefly, fucoidan and Dfuc were developed using a formic acid/n-butanol/water solvent and visualized with orcinol reagent. β-cyclodextrin, lactose and glucose were used as controls.

The Mw distribution of fucoidan and Dfuc was measured using an HPLC system with a TSK-GEL G5000PWXL column or a TSK-GEL G2500PWXL column [16]. The separation was performed using 0.1 M ammonium acetate, and the flow rate was maintained at 0.4 mL/min.

### 2.4. Chemical Composition Analysis

Total sugar content was measured by phenol-sulfuric acid method [17]. Protein content was assessed by Lowry method [18]. Sulfate group content was detected by BaCl_2_-gelatin method [19]. Uronic acid content was measured by carbazole method [20]. Functional groups were analyzed by a Fourier-transform infrared (FT-IR) spectrometer (Perkin Elmer, Shelton, CT, USA). Monosaccharide composition was determined using a pre-column derivatization method as previously described [21].

### 2.5. Animal Experiment

Animal experiment protocols were conducted in accordance with the National Research Council’s Guide for the Care and Use of Laboratory Animals and received approval from the Animal Ethics Committee of Dalian Polytechnic University (No. DLPU2023077). Male C57BL/6 mice (4 weeks old, SPF) were given free water and food in an SPF animal room (indoor temperature 23 ± 2 °C, humidity 50 ± 5%).

After acclimatization for a week, all mice were stratified according to their initial body weights and randomly divided into 4 groups (8 mice per group): normal chow diet (NC), high fat diet (HF), Fucoidan and Dfuc. All eight mice in each group were housed in a single cage. NC group was fed a normal chow diet (Research diets D12450B, 3.79 kcal/g), while HF, Fucoidan and Dfuc groups were fed a high-fat diet (Research diets D12492, 5.24 kcal/g). Meanwhile, Fucoidan and Dfuc groups were administered daily intragastric gavage of 300 mg/kg body weight of fucoidan or Dfuc, respectively, at approximately the same time for 10 consecutive weeks, while NC group and HF group were given the same volume of water.

During the experiment, food intake was recorded every other day at the cage level, and mice’s weight was measured weekly at a similar time of day. The estimated daily energy intake per mouse was calculated as follows: Mean daily energy intake (kcal/mouse/day) = [(food provided − food remaining) × diet energy density (kcal/g)]/[(number of mice per cage) × (number of days)]. The experiment lasted 10 weeks, and before it ended, fresh feces were collected separately from each mouse and stored at −80 °C. After fasting for 12 h following the last gavage, mice were sacrificed and blood samples, liver, epididymal adipose tissues, colon tissue were collected for further analysis.

### 2.6. Body Composition Analysis

Body composition of mice was observed using low-field nuclear magnetic resonance (Niumag Corporation, Suzhou, China) according to a reported method [10]. In the last week of the experiment, mice were anesthetized with isoflurane, placed into a properly sized scanning tube, and then scanned in the magnet cavity. Scanning parameters were as follows: cross-sectional imaging orientation, repetition time of 500 ms, echo time of 20 ms, and a cumulative scan count of 4.

### 2.7. Measurement of Biochemical Parameters

Blood samples were centrifuged (4 °C, 2000× *g*, 10 min) to separate serum. Liver tissue and colon tissue were separately added to normal saline and homogenized sufficiently to obtain 10% tissue homogenates. The levels of TC, TG, LDL-C, HDL-C, ALT, AST, MDA and T-SOD were determined by commercial assay kits (Nanjing Jiancheng, Jiangsu, China).

### 2.8. Histological Analysis

Fresh liver and distal colon (~1 cm from anus) were immersed in 4% paraformaldehyde solution. After fixation, the tissues were dehydrated using graded ethanol and subsequently embedded in paraffin. The tissues were cut into 6 μm slices before hematoxylin-eosin (H&E) staining. Finally, the slices were observed under an optical microscope. Histological evaluation was performed using coded sections by investigators blinded to the treatment groups. Hepatic steatosis and colonic histological injury were scored according to previously described methods [22] and [23], respectively.

### 2.9. Real-Time Quantitative PCR (RT-qPCR)

Total RNA was extracted from liver and colon tissues using RNAiso Plus and reverse-transcribed into cDNA using a PrimeScript RT reagent kit with gDNA Eraser(Takara Bio Inc., Kusatsu, Japan). RT-qPCR was performed in a 10 μL reaction system using TB Green Premix Ex Taq II and gene-specific primers (Table 1). The amplification conditions were as follows: pre-denaturation at 95 °C for 30 s, followed by 40 cycles of denaturation at 95 °C for 5 s and annealing/extension at 60 °C for 30 s. Melting curve analysis was performed to confirm primer specificity. β-actin was used as the reference gene, and relative quantification was normalized to β-actin by the 2^−ΔΔCt^ method.

### 2.10. 16S rRNA Gene Sequencing and Bioinformatics Analysis

Fresh feces were collected and their genomic DNA was isolated using a kit. Bacterial 16S rRNA gene V3-V4 region was amplified with primers 338F/806R. Sequencing was performed via the Illumina NovaSeq platform using the paired-end sequencing method. Raw reads were filtered and merged to obtain effective reads. The effective reads were clustered into operational taxonomic units (OTUs) at 97% sequence similarity. Representative OTU sequences were taxonomically assigned using a Bayesian classifier against the SILVA 138 database. Alpha diversity was evaluated using the ACE, Chao1, Shannon, and Simpson indices. Beta-diversity patterns were assessed using PCoA and UPGMA clustering based on Bray-Curtis distance. Differential bacterial taxa were identified using linear discriminant analysis effect size (LEfSe), with an LDA score >3.0. Bioinformatics analyses were conducted on the BMKCloud (https://www.biocloud.net/).

### 2.11. SCFAs Analysis

Fresh fecal samples (100 mg) were suspended in 400 μL saturated NaCl solution, followed by the addition of 20 μL 20% H_2_SO_4_ and thorough mixing using a vortex agitator. Next, 700 μL pre-cooled diethyl ether together with 100 μL internal standard solution (4 mM) was added, and the mixture was centrifuged (4 °C, 10,000× *g*, 15 min). Then, the upper organic layer was filtered through a 0.22 μm syringe filter. The concentrations of SCFAs were measured using a GC system equipped with a HP-INNOWAX column [24].

### 2.12. Targeted Metabolomics Analysis

Fresh fecal samples (100 mg) were mixed with 380 μL of pre-cooled acetonitrile/methanol/water solution (*v*/*v*/*v* = 2:2:1) and 20 μL of internal standard mixture. After standing at −30 °C for 4 h, these samples were centrifuged (13,800× *g*, 4 °C, 15 min) twice, and the supernatant was filtered through a membrane. The metabolites were separated by liquid chromatography (Shimadzu, Kyoto, Japan) equipped with a Waters BEH Amide column and analyzed by quadruple-linear ion trap (QTRAP) mass spectrometry in multiple reaction monitoring (MRM) mode. The chromatographic conditions and analytical procedures were detailed in our previous study [25]. Metabolomics data were analyzed using MetaboAnalyst 6.0 (https://www.metaboanalyst.ca/). The *p*-values obtained from comparisons of individual metabolites were adjusted using the Benjamini-Hochberg false discovery rate (FDR) procedure.

### 2.13. Statistical Analysis

Data are expressed as mean ± standard deviation. Homogeneity of variance was evaluated using Levene’s test. Weekly body weight was analyzed using a mixed-effects model, followed by separate Dunnett-adjusted comparisons with the HF and NC groups at each time point. Biochemical parameters were analyzed using one-way ANOVA, followed by Tukey’s HSD or Games-Howell post hoc tests, as appropriate. Spearman’s correlation analysis was performed, and the resulting *p*-values were adjusted using the FDR procedure. Statistical significance was defined as *p* < 0.05 or FDR-adjusted *p* < 0.05, as appropriate.

## 3. Results and Discussion

### 3.1. Chemical Characterizations

**Figure 1:**
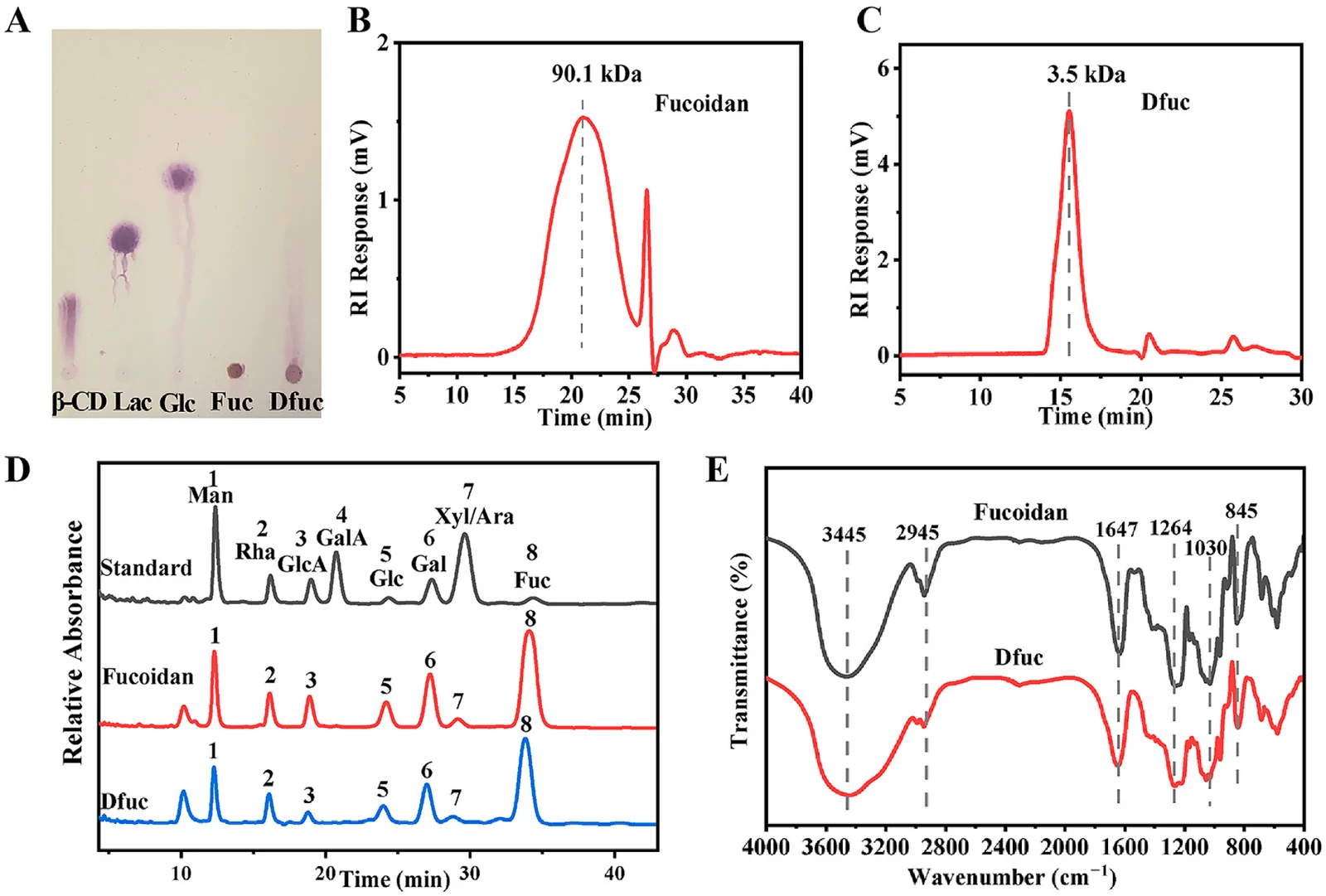
Structural characterization of fucoidan and Dfuc. TLC analysis (**A**), molecular weight distribution of fucoidan (**B**) and Dfuc (**C**), monosaccharide composition analysis (**D**), and FT-IR spectra (**E**).

To assess the impact of molecular weight reduction on the in vivo bioactivity of fucoidan under HFD conditions, depolymerized fucoidan (Dfuc) was prepared using photocatalytic degradation followed by ultrafiltration. TLC results showed that Dfuc contains oligosaccharides, unlike fucoidan (Figure 1A). The average molecular weight of fucoidan and Dfuc was 90.1 kDa and 3.5 kDa, respectively (Figure 1B,C). Chemical characteristics of fucoidan and Dfuc were similar, with both containing about 70% neutral sugars, less than 3% protein, and sulfate group content that did not decrease significantly. Uronic acid content of Dfuc was slightly lower (Table 2). Both fucoidan and Dfuc contain the same monosaccharides, mainly fucose, along with smaller amounts of mannose, galactose, glucose, rhamnose, xylose, arabinose, and glucuronic acid, though the ratios of these monosaccharides differ slightly (Table 2 and Figure 1D). Additionally, FT-IR spectra of fucoidan and Dfuc are similar (Figure 1E), showing no significant changes in functional groups. In conclusion, fucoidan and Dfuc have comparable chemical compositions and structural characteristics.

### 3.2. Fucoidan and Dfuc Alleviate HFD-Induced Obesity and Fat Accumulation

**Figure 2:**
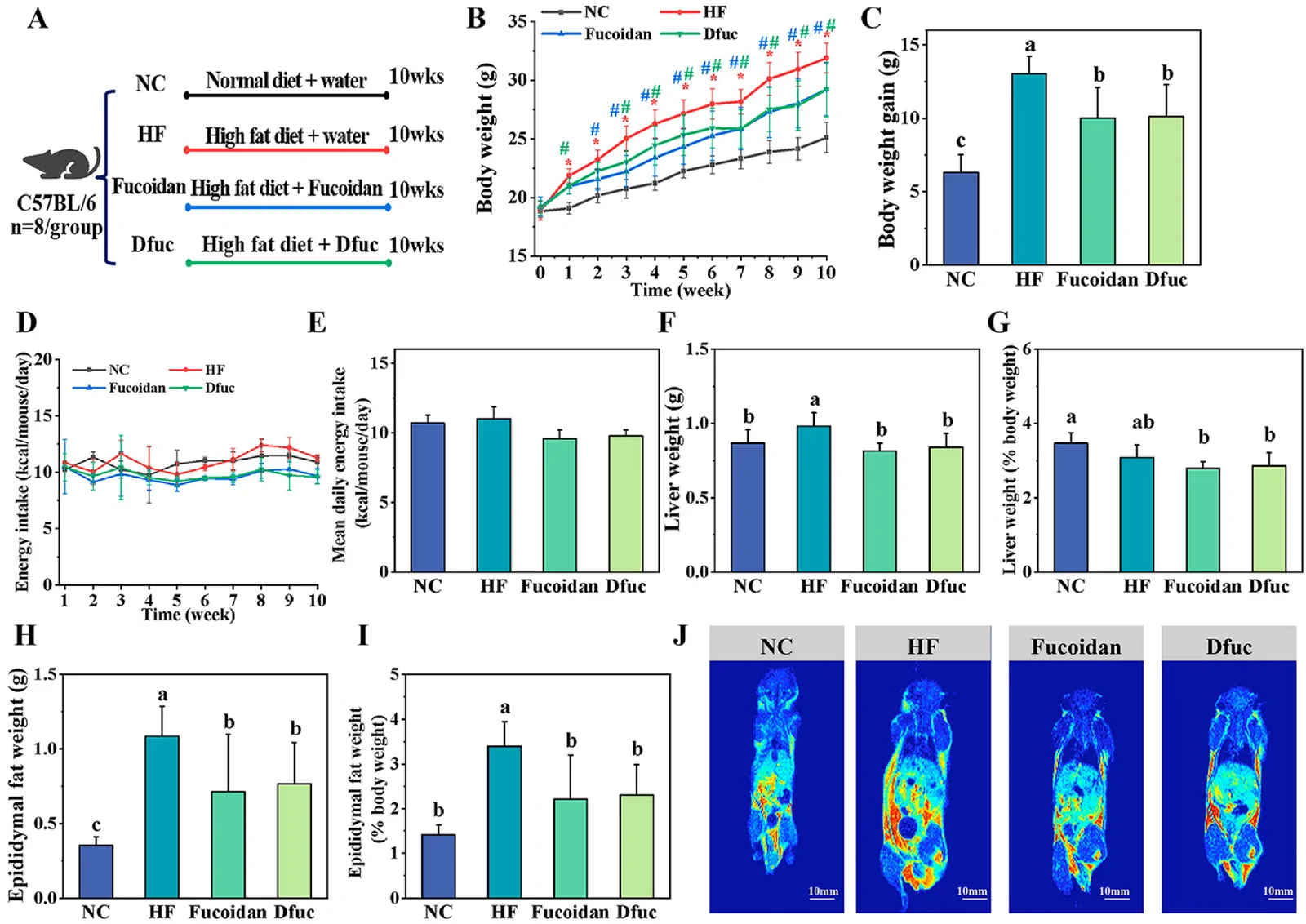
Effects of fucoidan and Dfuc on body weight and fat accumulation in HFD-fed mice. Experimental design (**A**), weekly body weight (**B**), body weight gain after 10 weeks of intervention (**C**), weekly estimated daily energy intake per mouse (**D**), mean estimated daily energy intake per mouse over the 10-week intervention period (**E**), liver weight (**F**), relative liver weight (% body weight) (**G**), epididymal fat weight (**H**), relative epididymal fat weight (% body weight) (**I**), and representative low-field NMR images (**J**). In the low-field NMR images, a deeper red color indicates a stronger fat-associated signal. For panels (**B**,**C**,**F**–**I**), data are presented as mean ± SD (*n* = 8 per group). Because food consumption was measured at the cage level with only one cage per group, no inferential statistical comparisons were performed for energy intake (**D**,**E**). NC, normal control group; HF, high-fat diet group; Fucoidan, fucoidan-treated group; Dfuc, depolymerized fucoidan-treated group. * *p* < 0.05 vs. NC group; blue #, *p* < 0.05 for Fucoidan vs. HF group; green #, *p* < 0.05 for Dfuc vs. HF group. Different letters represent the statistically significant differences (*p* < 0.05).

To evaluate the effects of fucoidan and Dfuc on obesity-related phenotypes, a HFD-induced obesity model was established in mice (Figure 2A). Compared to the NC group, the HF group exhibited significant increases in body weight and body weight gain. However, supplementation with fucoidan or Dfuc significantly attenuated these changes (Figure 2B,C). Additionally, daily energy intake appeared lower in Fucoidan and Dfuc groups than in HF group (Figure 2D,E). This observation is consistent with previous studies showing that fucoidans from *Ascophyllum nodosum* and *Laminaria japonica* reduce energy intake in HFD-fed mice, accompanied by attenuated obesity and improved hepatic steatosis [26]. These findings suggest that the reduced energy intake observed in the present study may partly contribute to the attenuation of body weight gain. Nevertheless, its exact relative contribution cannot be fully separated from direct biological effects in the absence of an HFD pair-fed control. Moreover, fucoidan and Dfuc significantly reduced liver, epididymal fat and relative index (% body weight) of epididymal fat in HFD-fed mice (Figure 2F–I). Representative low-field NMR imaging qualitatively illustrated weaker fat-associated signals in Fucoidan and Dfuc groups than in HF group (Figure 2J). These results indicate that fucoidan and Dfuc can ameliorate HFD-induced obesity and fat accumulation.

### 3.3. Fucoidan and Dfuc Improve HFD-Induced Hepatic Lipid Metabolic Abnormalities

HFD could induce dyslipidemia in both serum and hepatic tissues [2]. Compared with the NC group, HFD-fed mice exhibited significantly higher TC, TG, TC and LDL-C levels in serum and liver, indicating marked lipid metabolic abnormalities. However, fucoidan and Dfuc supplementation significantly reduced hepatic TC, TG and LDL-C levels and increased hepatic HDL-C levels, whereas no significant changes were observed in serum lipid parameters (Figure S1A,B). These results suggest that the lipid-modulating effects of fucoidan and Dfuc were more evident in liver under the present experimental conditions. Consistent with our findings, previous studies have shown that other polysaccharides, e.g., hawthorn fruit polysaccharides [27] and *Bangia fusco-purpurea* polysaccharide [28], can also reduce hepatic TC, TG and LDL-C levels and alleviate hepatic metabolic disorders. These findings indicate that fucoidan and Dfuc beneficially modulate hepatic lipid metabolism in HFD-fed mice, although no significant difference was observed between them.

### 3.4. Fucoidan and Dfuc Attenuate HFD-Induced Oxidative Stress and Chronic Inflammation in the Liver and Colon

**Figure 3:**
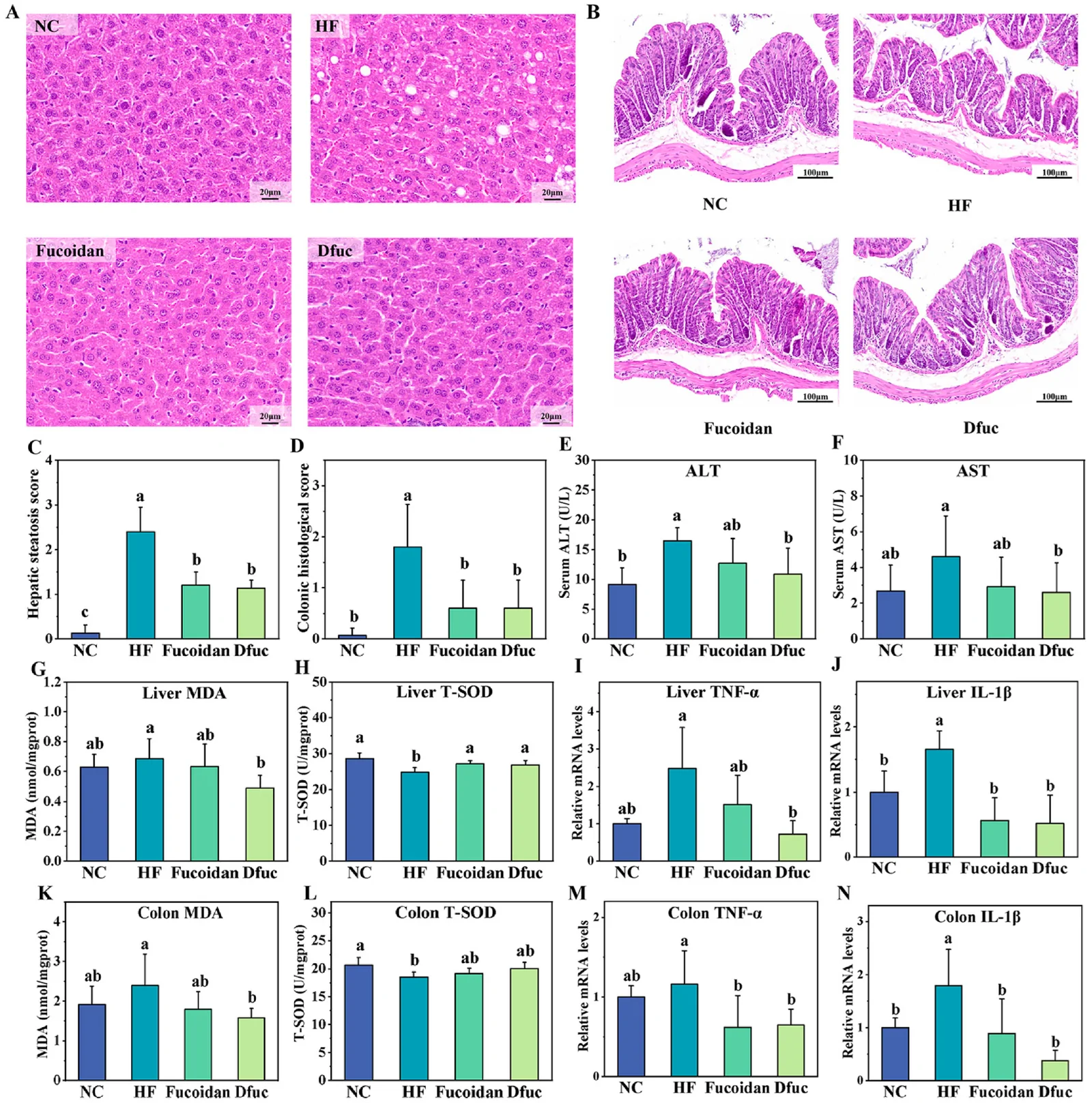
Effects of fucoidan and Dfuc on oxidative stress and chronic inflammation in the liver and colon of HFD-fed mice. Representative H&E staining images of the liver (**A**) and colon (**B**), liver steatosis score (**C**), colonic histological score (**D**), the levels of serum ALT (**E**) and AST (**F**), the levels of MDA (**G**) and T-SOD (**H**) in the liver, the relative expression of TNF-α (**I**) and IL-1β (**J**) in the liver, the levels of MDA (**K**) and T-SOD (**L**) in colon, and the relative expression of TNF-α (**M**) and IL-1β (**N**) in the colon. For histopathological scoring (**C**,**D**), *n* = 5 mice per group; for biochemical assays and gene expression analyses (**E**–**N**), *n* = 8 mice per group. Different letters represent the statistically significant differences (*p* < 0.05).

HFD-induced obesity is commonly accompanied by oxidative stress and chronic inflammation in the liver and colon [3]. Histological analysis revealed hepatic lipid droplet accumulation, and colonic mucosal injury in HFD-fed mice. However, supplementation with fucoidan or Dfuc effectively alleviated these pathological alterations (Figure 3A,B). Blinded semi-quantitative scoring confirmed that both fucoidan and Dfuc significantly reduced hepatic steatosis scores (Figure 3C) and colonic histological injury scores (Figure 3D). Serum ALT and AST, two common biomarkers of liver injury, were measured to evaluate hepatic damage [27]. ALT and AST levels were significantly increased in HF group, Dfuc supplementation significantly reduced both ALT and AST levels, whereas fucoidan exhibited a downward trend without statistical significance (Figure 3E,F). MDA and SOD are widely used indicators of lipid peroxidation and antioxidant capacity [29]. In the liver, both fucoidan and Dfuc significantly increased SOD activity (Figure 3H), while Dfuc significantly reduced MDA levels compared to HF group (Figure 3G). In the colon, Dfuc supplementation significantly decreased MDA levels (Figure 3K), whereas SOD activity in both treatment groups showed an increasing trend compared to HF group without reaching statistical significance (Figure 3L). Moreover, inflammatory responses were evaluated by determining the relative expression of TNF-α and IL-1β. In the liver, both fucoidan and Dfuc significantly reduced IL-1β expression, while Dfuc markedly suppressed TNF-α expression (Figure 3I,J). In the colon, both treatments significantly reduced TNF-α expression, and Dfuc also significantly decreased IL-1β expression (Figure 3M,N). These results indicate that fucoidan and Dfuc alleviated HFD-induced hepatic and colonic oxidative stress and inflammation to varying degrees, with Dfuc exhibiting significant improvements in selected oxidative and inflammatory markers (such as AST, MDA, and TNF-α) compared with HF group.

### 3.5. Fucoidan and Dfuc Modulate HFD-Induced Gut Microbiota Dysbiosis

**Figure 4:**
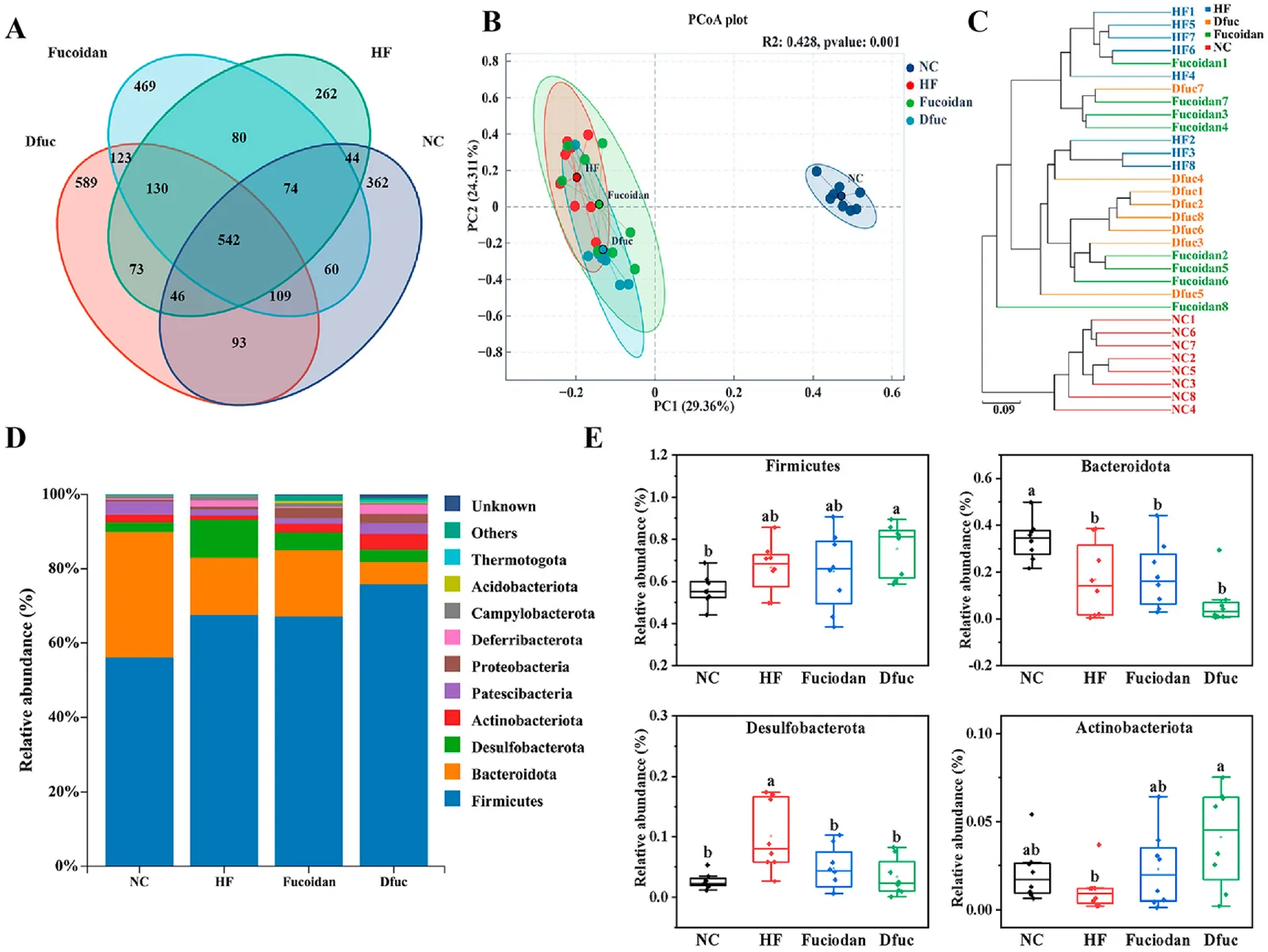
Effects of fucoidan and Dfuc on HFD-induced gut microbiota dysbiosis (*n* = 8). Venn diagram of OTUs (**A**), PCoA plot with group centroids (larger black-bordered markers) (**B**), and UPGMA clustering tree (**C**) based on Bray–Curtis distance on OTUs, bacterial taxonomic profiling at the phylum (**D**), and differences in the relative abundances of Firmicutes, Bacteroidota, Desulfobacterota and Actinobacteriota (**E**). Different letters represent the statistically significant differences (*p* < 0.05).

To further evaluate the effects of fucoidan and Dfuc on this alteration, gut microbial composition was analyzed using 16S rRNA gene sequencing. After quality filtering to remove low-quality reads, an average of 72283 ± 2310 effective reads per sample was obtained. The average sequence lengths for NC, HF, Fucoidan, and Dfuc groups were 420 ±3 bp, 416 ± 2 bp, 417 ± 2 bp, and 420 ± 3 bp, respectively. The sequencing effective ratio exceeded 80% across all samples (Table S1), indicating adequate sequencing depth and high data quality for downstream bioinformatics analysis. As shown in Figure S2, supplementation with fucoidan or Dfuc had only minor effects on the ACE, Chao1, Shannon, and Simpson indices, indicating a limited influence on the overall bacterial richness and diversity. However, Venn diagram analysis, PCoA with PERMANOVA (R^2^ = 0.428, *p* = 0.001), and UPGMA clustering revealed clear differences and distinct clustering patterns among different groups (Figure 4A–C), suggesting that fucoidan and Dfuc markedly modulate the structural composition of the gut microbiota. In addition, gut microbial composition at phylum level is presented in Figure 4D,E. Compared with NC group, HF group showed increased relative abundances of Firmicutes and Desulfobacterota and decreased Bacteroidetes and Actinobacteriota. Fucoidan restored Desulfobacterota and Actinobacteriota to levels similar to those in the NC group, without significantly affecting Firmicutes or Bacteroidetes. In comparison, Dfuc increased Firmicutes and Actinobacteriota and reduced Desulfobacterota, while Bacteroidetes remained unchanged.

**Figure 5:**
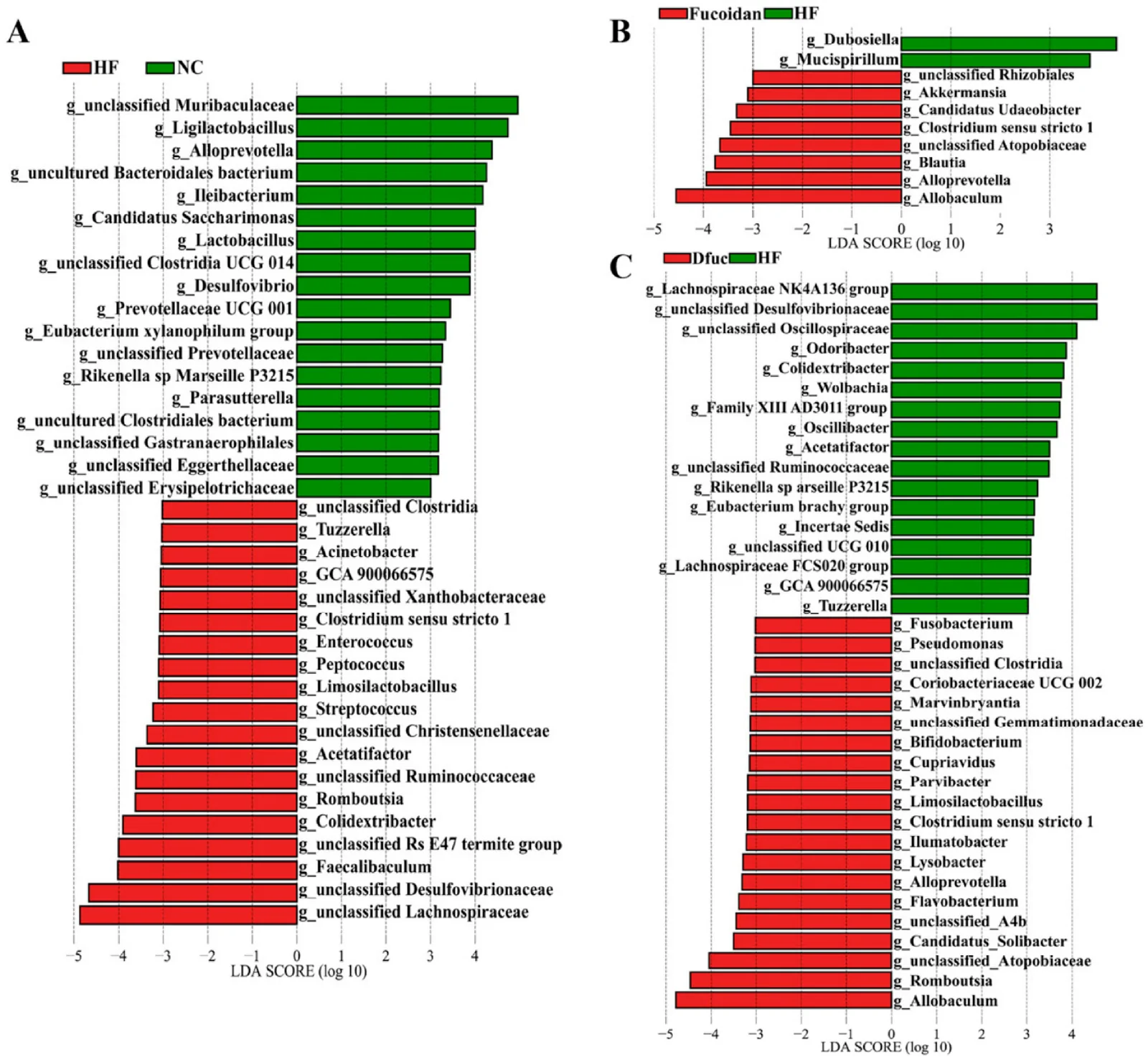
LEfSe analysis (LDA score > 3.0) between NC and HF groups (**A**), between Fucoidan and HF groups (**B**), and between Dfuc and HF groups (**C**).

To further explore the microbial composition alterations, LEfSe (LDA > 3.0) analysis was performed to identify differential taxa at the genus level (Figure 5). Compared with the NC group, the relative abundances of *Ligilactobacillus*, *Alloprevotella*, *Lactobacillus*, and *Prevotellaceae* UCG-001 were significantly reduced in the HF group, whereas unclassified Lachnospiraceae, unclassified Desulfovibrionaceae, *Enterococcus*, and *Faecalibaculum* were significantly enriched. However, fucoidan treatment markedly increased the levels of *Alloprevotella*, *Allobaculum*, *Akkermansia*, and *Blautia* relative to HF group. Dfuc group showed a significant enrichment of *Allobaculum*, *Alloprevotella*, *Romboutsia*, *Flavobacterium*, *Bifidobacterium* and *Fusobacterium*.

*Alloprevotella*, belonging to the phylum Bacteroidetes, is considered a health-associated genus due to its ability to ferment complex polysaccharides into SCFAs, thereby protecting intestinal health and suppressing inflammatory responses [30]. In the present study, the relative abundance of *Alloprevotella* was reduced in the HF group, while fucoidan or Dfuc treatment significantly restored its levels in the intestine. Previous studies have shown that *Cordyceps militaris* polysaccharides prevent obesity in HFD-fed mice, accompanied by an increase in *Alloprevotella* abundance [31]. These findings imply that the increased abundance of *Alloprevotella* may be associated with the anti-obesity effects of fucoidan and Dfuc. In addition, administration of fucoidan and Dfuc promoted enrichment of *Allobaculum* in gut microbiota. Previous literature has indicated that *Allobaculum* produces beneficial metabolites like SCFAs, and it plays an important role in improving metabolic health and maintaining intestinal barrier function, which may help mitigate HFD-induced obesity [32]. Thus, enrichment of *Allobaculum* may also be associated with the beneficial effects of fucoidan and Dfuc on gut microbial balance. Besides, fucoidan significantly increased *Akkermansia* and *Blautia*. *Akkermansia* is a mucin-degrading bacterium that contributes to maintaining intestinal barrier integrity and has been widely linked to improved metabolic profiles in obesity models [8]. *Blautia* is a short-chain fatty acid-producing genus, and recent evidence indicates that it supports colonic mucus function and barrier maintenance, particularly under dietary stress conditions [33]. Meanwhile, Dfuc increased *Romboutsia* and *Bifidobacterium*. *Romboutsia* has been associated with carbohydrate metabolism and SCFAs production, contributing to metabolic homeostasis under high-fat diet conditions [34]. *Bifidobacterium* is a commonly used probiotic genus that contributes to maintaining gut microbial homeostasis and gastrointestinal function [3]. In the present study, Dfuc markedly enriched *Bifidobacterium* in HFD-fed mice. This enrichment may be attributed to the oligosaccharide structures present in Dfuc, which can serve as fermentable substrates to stimulate *Bifidobacterium* growth. Similar studies have shown that several seaweed-derived oligosaccharides, such as alginate oligosaccharides [35] and agar oligosaccharides [36], effectively promote the proliferation of *Bifidobacterium*. Taken together, these findings indicate that fucoidan and Dfuc can alleviate HFD-induced gut microbiota dysbiosis to varying extents through modulation of distinct bacterial taxa.

### 3.6. Fucoidan and Dfuc Regulate HFD-Induced Microbial Metabolism

**Figure 6:**
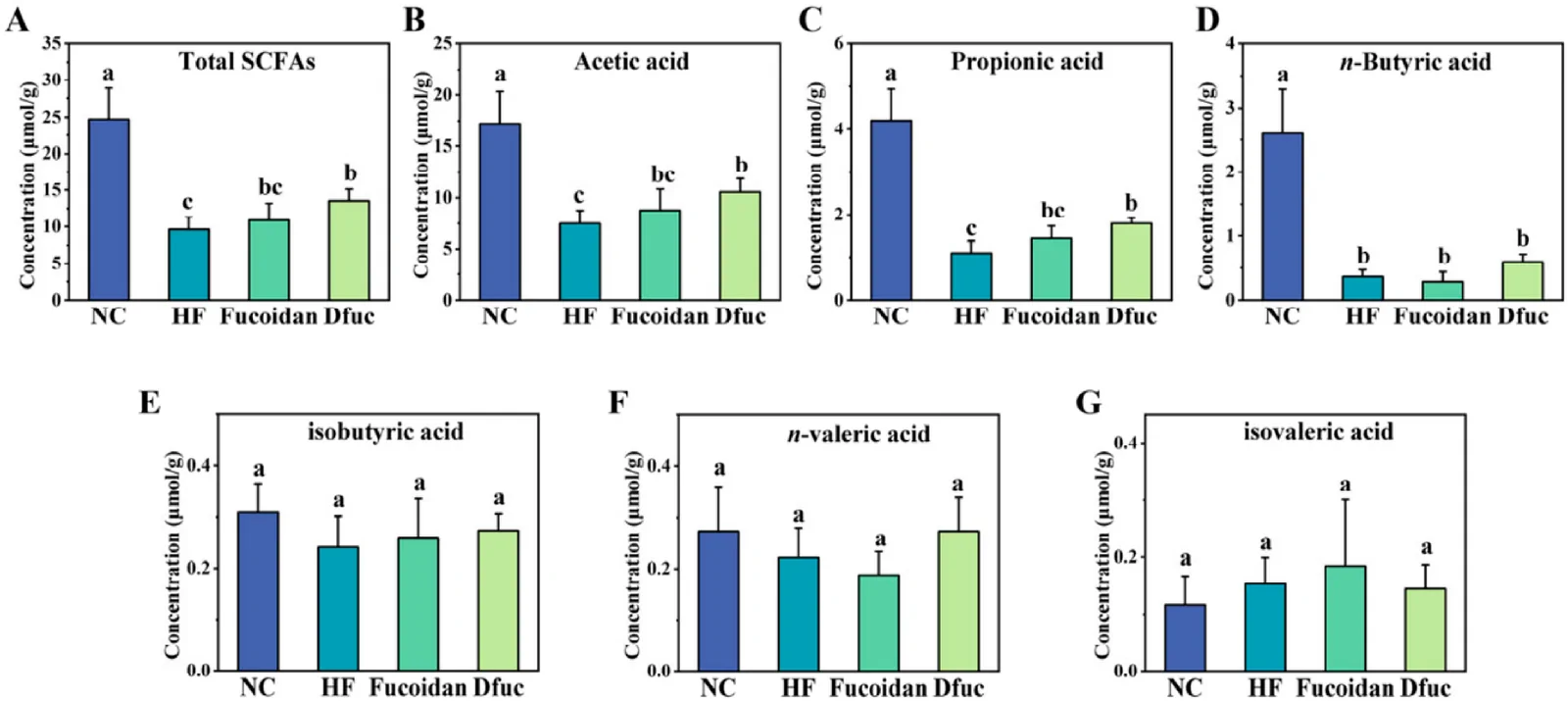
Effects of fucoidan and Dfuc on fecal SCFA concentrations (*n* = 8). Total SCFAs (**A**), acetic acid (**B**), propionic acid (**C**), *n*-butyric acid (**D**), isobutyric acid (**E**), *n*-valeric acid (**F**) and isovaleric acid (**G**). Different letters represent the statistically significant differences (*p* < 0.05).

SCFAs are major metabolites produced by gut microbial fermentation of polysaccharides and play essential roles in maintaining mucosal barrier integrity, regulating immune responses, and providing energy to intestinal epithelial cells [37]. As shown in Figure 6, the levels of acetate, propionate, butyrate, and total SCFAs in feces were significantly reduced in the HF group compared with the NC group. However, Dfuc supplementation significantly increased fecal concentrations of total SCFAs, acetate, and propionate compared to HF group (*p* < 0.05), whereas fucoidan exhibited an upward trend without statistical significance (Figure 6A–C). Neither treatment had a significant effect on fecal butyrate levels (Figure 6D). These findings are in agreement with our previous in vitro fermentation results, which demonstrated that both fucoidan and its degradation product enhance the accumulation of acetate and propionate [13]. Notably, Dfuc exerted a marked restoring effect on fecal acetate and propionate concentrations under high-fat diet conditions. Previous studies have reported that low-molecular-weight polysaccharides can be more readily utilized by intestinal microbiota, thereby promoting SCFA accumulation [38]. In addition, Xu et al. [9] reported that oligosaccharides from *Gracilaria lemaneiformis* more effectively increased SCFA production and improved the intestinal microenvironment in HFD-fed mice. These results suggest that fucoidan and Dfuc partially restore HFD-induced alterations in fecal SCFA profiles by boosting acetate and propionate levels, with Dfuc showing a significant improvement relative to HF group.

**Figure 7:**
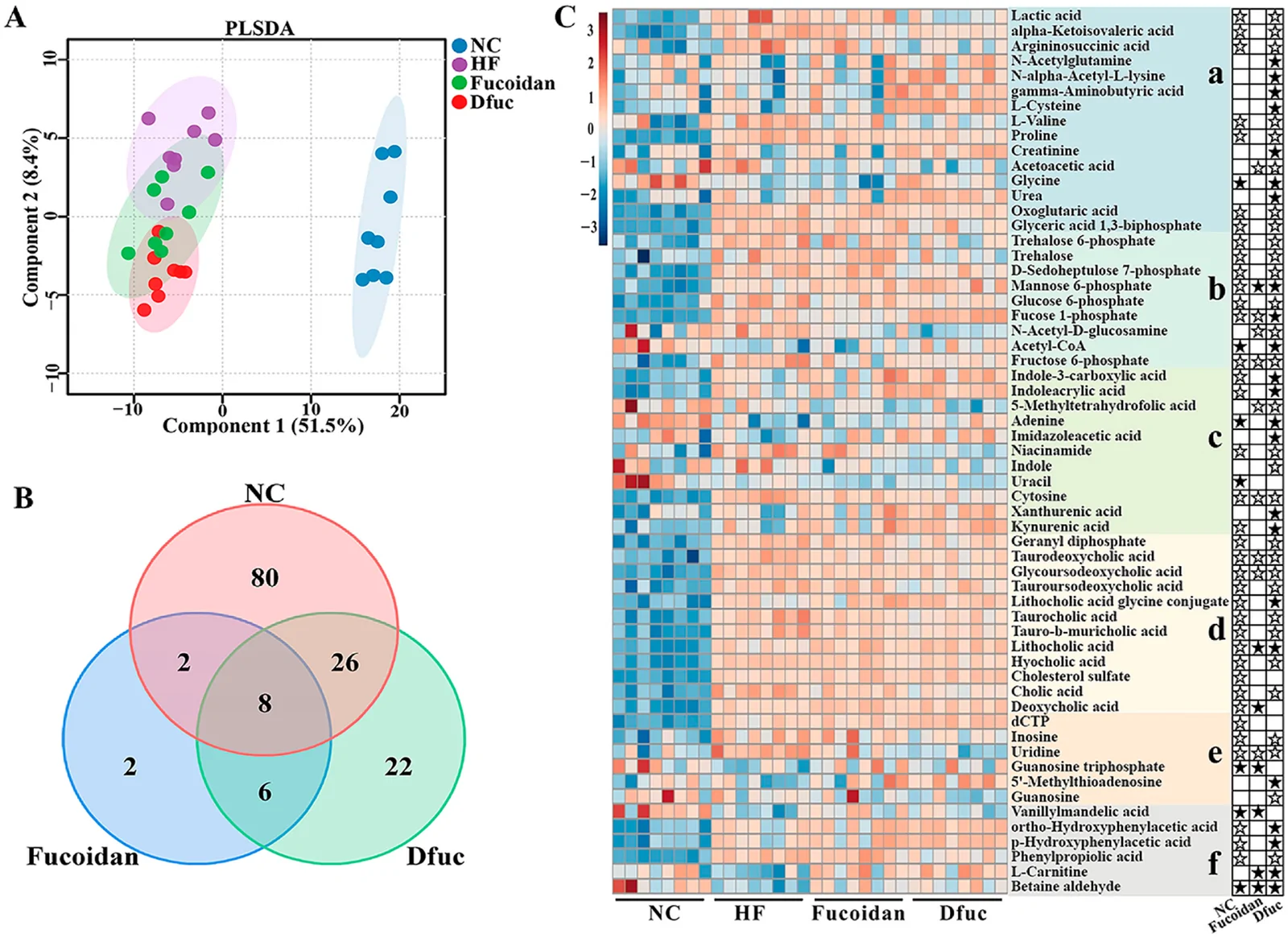
Analysis of metabolite profiles (*n* = 8). PLS-DA analysis (**A**), venn plot among NC, Fucoidan, and Dfuc groups compared to HF group (FDR-adjusted *p* < 0.05, VIP > 1) (**B**), and heatmap of the differential metabolites (**C**); classifications of significantly differential metabolites in organic acids and derivatives (**a**), organic oxygen compounds (**b**), organoheterocyclic compounds (**c**), lipids and lipid-like molecules (**d**), nucleosides, nucleotides, and analogues (**e**), and others (**f**). “★” and “☆” mean more and less abundant in other groups compared with HF group (FDR-adjusted *p* < 0.05), respectively.

To further investigate the effects of fucoidan and Dfuc on microbial metabolites, fecal metabolic profiles were examined using targeted HPLC-MS/MS metabolomics. PLS-DA analysis revealed a clear separation of NC group from HF, fucoidan, and Dfuc groups (Figure 7A). The HF group was also distinctly separated from the Dfuc group, whereas the Fucoidan group was located between the HF and Dfuc groups, with no obvious separation from either group. Further, OPLS-DA analysis showed distinct metabolic profiles between HF group and NC, fucoidan, and Dfuc groups (Figure S2). Using VIP > 1 and FDR-adjusted *p* < 0.05 as criteria, a total of 196 differential metabolites were identified. Among them, 116, 18, and 62 metabolites were identified in NC, Fucoidan, and Dfuc groups, respectively (Figure 7B). These results suggest that fucoidan and Dfuc altered gut microbial metabolism to different extents.

Subsequently, differential metabolites were filtered using fold change (FC) ≥1.5 or ≤0.67 for heatmap analysis. As illustrated in Figure 7C, metabolite alterations were mainly distributed in organic acids and derivatives (25.4%), organooxygen compounds (15.3%), organic heterocyclic compounds (18.6%), lipids and lipid-like molecules (20.3%), and nucleosides, nucleotides, and analogues (10.2%). Compared with the HF group, the Fucoidan group showed 7 increased and 9 decreased metabolites, whereas the Dfuc group showed 23 increased and 30 decreased metabolites. These findings indicate a broader regulatory effect of Dfuc on HFD-induced metabolic alterations.

Among these metabolites, acetoacetic acid is associated with amino acid and fatty acid metabolism, and HFD can increase intestinal acetoacetic acid levels, thereby contributing to oxidative damage [39]. In the present study, both fucoidan and Dfuc reduced acetoacetic acid levels. In addition, valine, proline, and lactic acid have been reported to be positively associated with obesity and related metabolic disorders [40,41], whereas glycine levels are reduced in patients with obesity and cardiovascular diseases [42]. Notably, Dfuc decreased valine, proline, and lactic acid levels while increasing glycine levels, suggesting that it may more effectively reduce the risk of HFD-induced metabolic disorders through modulating amino acid-related metabolites. Moreover, HFD is also known to disrupt carbohydrate metabolism, including glycolysis/gluconeogenesis, pentose phosphate pathway, and tricarboxylic acid cycle, etc. [43]. In the present study, fucoidan and Dfuc both increased mannose 6-phosphate levels, while decreasing fructose 6-phosphate and N-acetyl-D-glucosamine levels to different extents. Dfuc regulated a broader range of carbohydrate metabolism-related metabolites, including acetyl-CoA, glucose 6-phosphate, and D-sedoheptulose 7-phosphate. Bile acid metabolism is a key mechanism involved in cholesterol homeostasis, and excessive fat intake, including cholesterol, can disturb bile acid metabolite balances [44]. In this study, both fucoidan and Dfuc reduced taurodeoxycholic acid and glycoursodeoxycholic acid levels while increasing lithocholic acid levels. In addition, fucoidan increased deoxycholic acid levels, whereas Dfuc increased lithocholic acid glycine conjugate and decreased tauroursodeoxycholic acid and tauro-β-muricholic acid levels. These findings suggest that both fucoidan and Dfuc may modulate bile acid-related metabolite profiles. This is consistent with the study by You et al. [45], who reported that sulfated polysaccharides from *Caulerpa lentillifera* reduced fecal bile acids and altered bile acid profiles in HFD-fed mice. Moreover, both fucoidan and Dfuc reduced uridine levels while increasing L-carnitine and betaine aldehyde levels. Elevated uridine levels have been reported in obese patients, supporting its association with obesity-related metabolic disturbances [46]. L-carnitine and betaine aldehyde have been associated with improved antioxidant status and attenuation of intestinal oxidative stress [47,48]. Taken together, fucoidan and Dfuc alleviated HFD-induced metabolic disturbances mainly by modulating metabolite profiles associated with amino acid, carbohydrate, and bile acid metabolic pathways, thereby contributing to an improved intestinal metabolic environment. In addition, Dfuc influenced a more diverse panel of metabolites than fucoidan, suggesting that reduced molecular weight may be associated with broader alterations in gut microbiota-related metabolic profiles.

### 3.7. Correlations Among Gut Microbiota, Metabolites, and Obesity-Related Parameters

**Figure 8:**
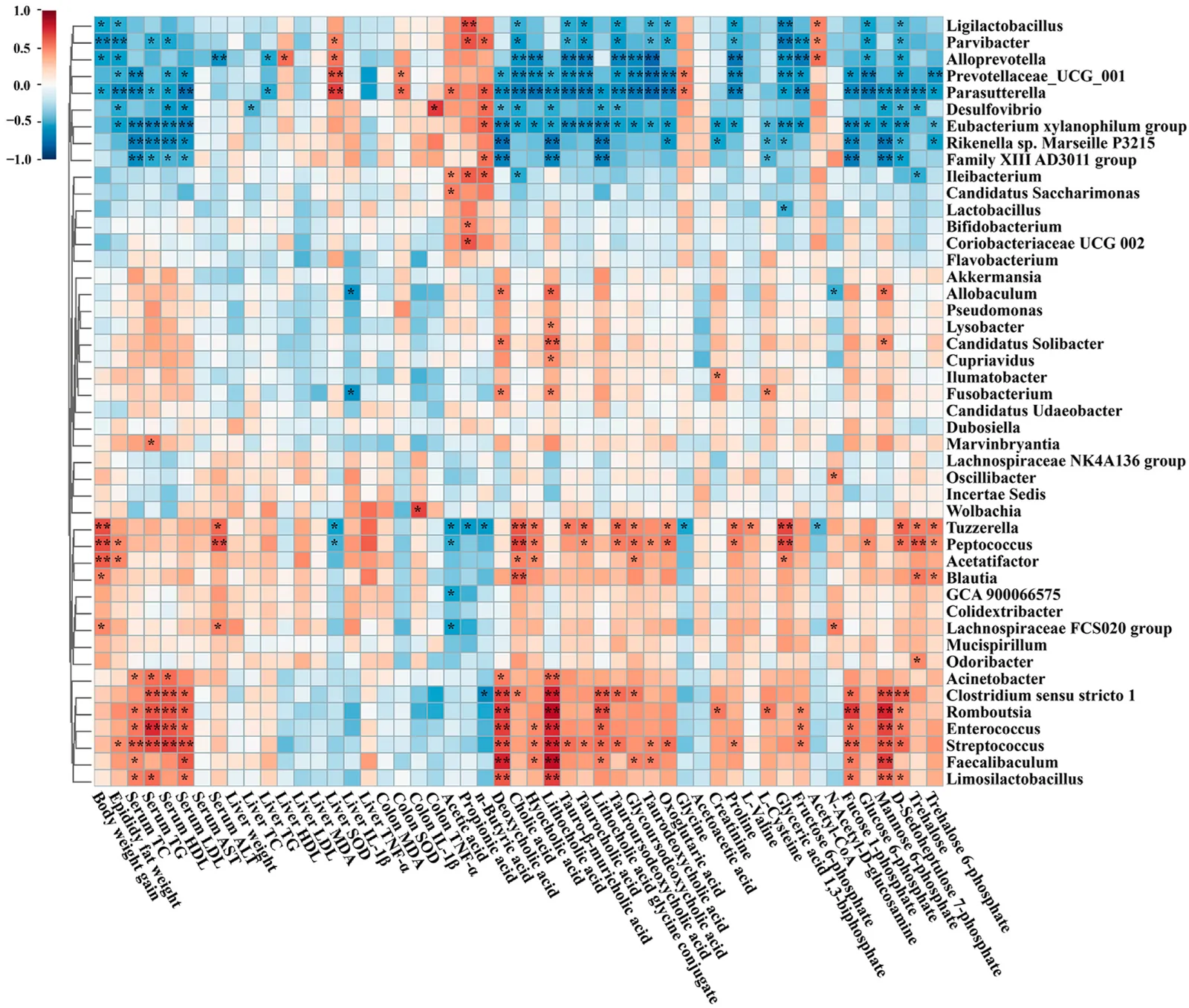
Heatmap of Spearman’s correlation between key bacterial genera, obesity-related parameters, fecal SCFAs, and differential metabolites. Key bacterial genera were selected based on the LEfSe results (LDA score > 3.0). The color scale represents Spearman’s correlation coefficient (r), with red indicating positive correlations and blue indicating negative correlations. The resulting p-values were adjusted using the FDR procedure. * FDR-adjusted *p* < 0.05 (*q* < 0.05) and ** FDR-adjusted *p* < 0.01 (*q* < 0.01).

To explore the potential associations among gut microbiota, metabolites, and obesity-related parameters, Spearman’s correlation analysis was performed (Table S2). Key bacterial genera, obesity-related parameters, fecal SCFA concentrations, and representative differential fecal metabolites were included in the correlation matrix (Figure 8). Overall, several bacterial genera showed varying degrees of correlation with these parameters and metabolites, suggesting close associations between gut microbial shifts and HFD-induced metabolic alterations. Notably, *Alloprevotella*, which was decreased in the HF group and restored by fucoidan and Dfuc intervention, displayed broad correlations with obesity-related parameters and fecal metabolites. *Alloprevotella* was negatively correlated with body weight gain, epididymal fat weight, serum ALT, liver TG, and several metabolites related to bile acid, amino acid, and carbohydrate metabolism including taurodeoxycholic acid, proline, and fructose 6-phosphate. In contrast, it was positively correlated with hepatic SOD and acetyl-CoA. These results suggest that the restoration of *Alloprevotella* may be associated with attenuation of HFD-induced metabolic disturbances. In addition, *Allobaculum*, another genus enriched in both fucoidan and Dfuc groups, was negatively correlated with liver IL-1β and N-acetyl-D-glucosamine and positively correlated with mannose 6-phosphate, deoxycholic acid, and lithocholic acid, suggesting that its abundance co-varies with host inflammatory status and microbial metabolic profiles. Moreover, *Bifidobacterium*, which was significantly enriched in Dfuc group, was only positively correlated with fecal propionate concentration, whereas *Akkermansia*, specifically enriched in Fucoidan group, did not reach FDR significance (*q* > 0.05), but showed negative correlation trends with N-acetyl-D-glucosamine and positive trends with deoxycholic acid at the unadjusted level (*p* < 0.05, Table S2). Importantly, these correlation analyses describe statistical co-variations rather than definitive causality; direct metabolite production and causal mechanisms warrant further validation through mono-colonization or in vitro fermentation models. These findings indicate that the beneficial effects of fucoidan and Dfuc on HFD-induced metabolic disturbances are closely associated with coordinated changes in gut microbiota composition and fecal metabolic profiles.

## 4. Conclusions

The results showed that fucoidan and Dfuc both alleviated HFD-induced obesity and fat accumulation in mice, accompanied by improvements in oxidative status and inflammatory responses in the liver and colon. In particular, Dfuc exhibited significant beneficial effects on selected oxidative stress and inflammatory markers compared with the HF group. In addition, fucoidan and Dfuc differentially altered gut microbiota composition, fecal SCFA concentrations, and metabolic profiles. Notably, Dfuc significantly elevated fecal acetate and propionate concentrations and induced broader shifts in metabolites related to amino acid, carbohydrate, and bile acid metabolism. These findings suggest that Dfuc has promising potential to exert anti-obesity and gut microbiota-related metabolic regulatory effects.

## Figures and Tables

**Table 1:** Sequences of primers used in quantitative PCR.

Gene	Forward Primer (5′→3′)	Reverse Primer (5′→3′)
β-actin	TCAGCAAGCAGGAGTACGATG	AACGCAGCTCAGTAACAGTCC
TNF-α	ATGAGCACAGAAAGCATGATC	TACAGGCTTGTCACTCGAATT
IL-1β	TTCATCTTTGAAGAAGAGCCCAT	TCGGAGCCTGTAGTGCAGTT

**Table 2:** Chemical characteristics of fucoidan and Dfuc.

Chemical Composition	Fucoidan	Dfuc
Neutral sugars (%)	70.7 ± 0.6 ^a^	75.1 ± 9.3 ^a^
Protein (%)	2.81 ± 0.35 ^a^	1.77 ± 0.03 ^b^
Sulfate group (%)	27.1 ± 1.9 ^a^	25.1 ± 0.3 ^a^
Uronic acid content (%)	16.9 ± 0.6 ^a^	12.9 ± 0.4 ^b^
Main monosaccharides (molar ratio)		
Fuc	8.7	8.4
Gal	2.6	2.2
Man	1.6	1.4
Glc	1.6	1.4
Rha	1.0	1.0

Note: Different superscript letters represent the statistically significant differences (*p* < 0.05). Molar ratio is expressed relative to Rha. Fuc: fucose; Gal: galactose; Man: mannose; Glc: glucose; Rha: rhamnose.

## Data Availability

The original contributions presented in this study are included in the article/Supplementary Materials. Further inquiries can be directed to the corresponding author.

## Supplementary Materials

The following supporting information can be downloaded at: https://www.mdpi.com/article/10.3390/foods15193508/s1, Figure S1: Effects of fucoidan and Dfuc on serum and hepatic lipid parameters in HFD-fed mice (*n* = 8); Figure S2: Alpha diversity analysis (*n* = 8); Figure S3: OPLS-DA analysis and permutation tests among NC, HF, Fucoidan and Dfuc groups (*n* = 8); Table S1: 16S rRNA gene data preprocessing statistics and quality control; Table S2: Spearman’s correlation coefficients (r) and FDR-adjusted *p*-values (q) between key bacterial genera, obesity-related parameters, fecal SCFAs, and differential metabolites.
