# Supplementary material for: Depolymerized Fucoidan Alleviates High-Fat Diet-Induced Obesity in Association with Alterations in Gut Microbiota and Metabolic Profiles
Source: Foods. 2026 Oct 1;15(19):3508. doi: 10.3390/foods15193508 (PMC13665278; doi:10.3390/foods15193508)
Supplement: Supplementary file 1 [file foods-15-03508-s001.zip › Supplementary data.pdf]

# Depolymerized Fucoidan Alleviates High-Fat Diet-Induced Obesity in Association with Alterations in Gut Microbiota and Metabolic Profiles

Xiaona Sun [1,2], Lin Bai [2], Changyu Su [2], Chunqing Ai [2], Xiaomeng Ren [2], Fanhua Kong [2], and Shuang Song [2,*]

1 School of Smart New Agricultural Industry, Dalian Art College, Dalian 116600, P. R. China

2 SKL of Marine Food Processing & Safety Control, National Engineering Research Center of Seafood, Collaborative Innovation Center of Seafood Deep Processing, National & Local Joint Engineering Laboratory for Marine Bioactive Polysaccharide Development and Application, Liaoning Key Laboratory of Food Nutrition and Health, School of Food Science and Technology, Dalian Polytechnic University, Dalian 116034, China

* Correspondence: songs1008@163.com

# Supplementary materials

**Supplemental Figure**

**Figure S1:** Effects of fucoidan and Dfuc on serum and hepatic lipid parameters in HFD-fed mice (n=8). Serum levels of TC (A), TG (B), LDL-C (C), and HDL-C (D), and hepatic levels of TC (E), TG (F), LDL-C (G), and HDL-C (H). Different letters indicate significant differences among groups ($p < 0.05$).

**Figure S2**: Alpha diversity analysis (n=8). Different letters indicate significant differences among groups ($p < 0.05$).

**Figure S3**: OPLS-DA analysis and permutation tests among NC, HF, Fucoidan and Dfuc groups (n=8).

**Supplemental Table**

**Table S1:** 16S rRNA data preprocessing statistics and quality control

## Figure S1

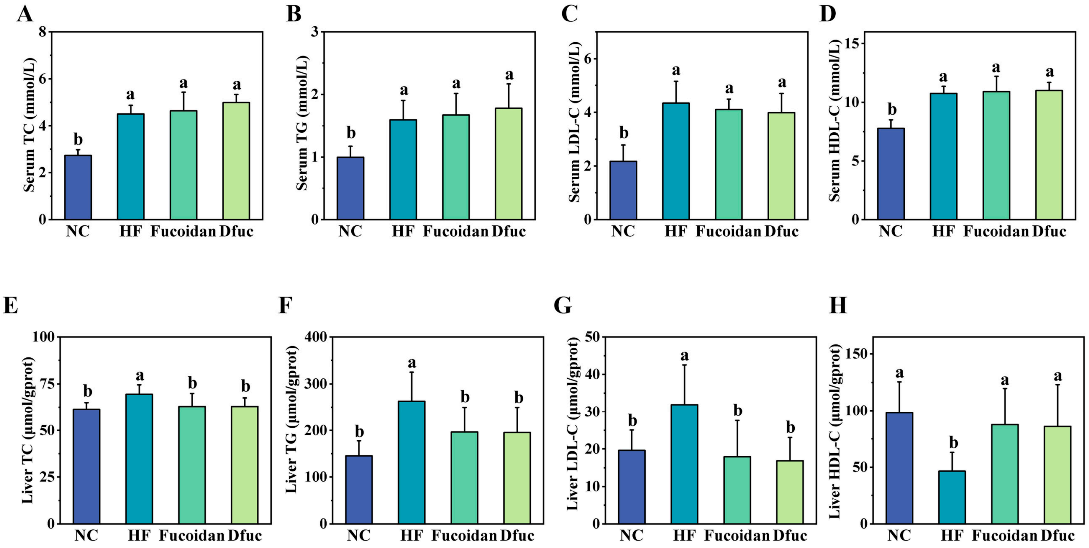
A
B
C
D
E
F
G
H
Serum TC (mmol/L)
Serum TG (mmol/L)
Serum LDL-C (mmol/L)
Serum HDL-C (mmol/L)
Liver TC (μmol/gprot)
Liver TG (μmol/gprot)
Liver LDL-C (μmol/gprot)
Liver HDL-C (μmol/gprot)
NC
HF
Fucoidan
Dfuc

# Figure S2

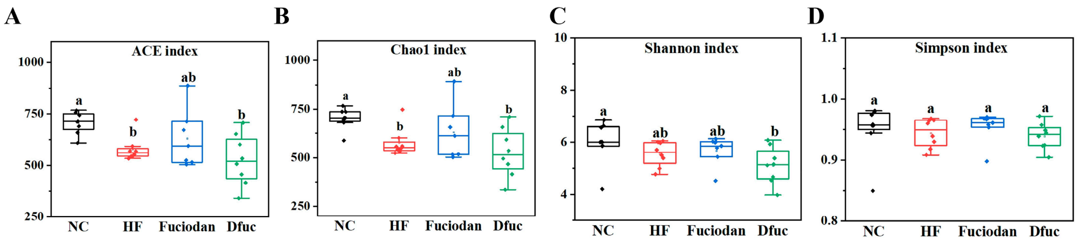
A
ACE index
1000
750
500
250
a
b
ab
b
NC
HF
Fuciodan
Dfuc
B
Chao1 index
1000
750
500
250
a
b
ab
b
NC
HF
Fuciodan
Dfuc
C
Shannon index
10
8
6
4
a
ab
ab
b
NC
HF
Fuciodan
Dfuc
D
Simpson index
1.1
1.0
0.9
0.8
a
a
a
a
NC
HF
Fuciodan
Dfuc

# Figure S3

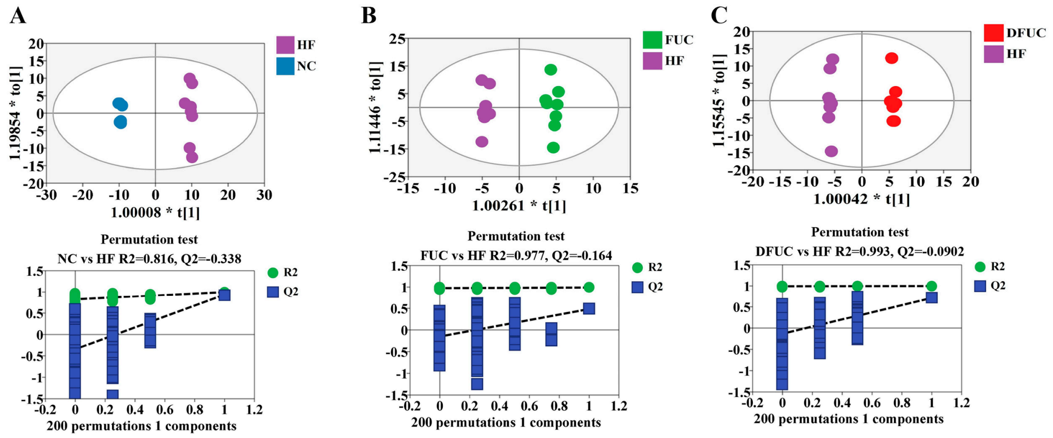
A
HF
NC
1.19854 * to[1]
1.00008 * t[1]
Permutation test
NC vs HF R2=0.816, Q2=-0.338
R2
Q2
200 permutations 1 components
B
FUC
HF
1.11446 * to[1]
1.00261 * t[1]
Permutation test
FUC vs HF R2=0.977, Q2=-0.164
R2
Q2
200 permutations 1 components
C
DFUC
HF
1.15545 * to[1]
1.00042 * t[1]
Permutation test
DFUC vs HF R2=0.993, Q2=-0.0902
R2
Q2
200 permutations 1 components

**Table S1** 16S rRNA gene data preprocessing statistics and quality control

| Sample | Raw Reads | Clean Reads | Effective Reads | AvgLen (bp) | Effective (%) |
|---|---|---|---|---|---|
| NC1 | 80199 | 75089 | 72508 | 417 | 90.41 |
| NC2 | 80346 | 75273 | 74480 | 416 | 92.7 |
| NC3 | 79887 | 74437 | 71954 | 422 | 90.07 |
| NC4 | 79939 | 74306 | 70856 | 425 | 88.64 |
| NC5 | 80303 | 74682 | 73464 | 418 | 91.48 |
| NC6 | 80151 | 75027 | 73435 | 421 | 91.62 |
| NC7 | 79773 | 73956 | 71105 | 420 | 89.13 |
| NC8 | 79885 | 73851 | 72521 | 422 | 90.78 |
| HF1 | 79846 | 74655 | 72714 | 417 | 91.07 |
| HF2 | 80023 | 74543 | 73907 | 416 | 92.36 |
| HF3 | 80101 | 75445 | 73503 | 416 | 91.76 |
| HF4 | 80006 | 75210 | 72048 | 413 | 90.05 |
| HF5 | 80016 | 74255 | 68038 | 419 | 85.03 |
| HF6 | 80273 | 74868 | 71201 | 414 | 88.7 |
| HF7 | 80002 | 74574 | 66136 | 419 | 82.67 |
| HF8 | 80237 | 75541 | 74238 | 414 | 92.52 |
| Fucoidan1 | 80020 | 74950 | 71352 | 418 | 89.17 |
| Fucoidan 2 | 80143 | 75082 | 74194 | 418 | 92.58 |
| Fucoidan 3 | 80022 | 74448 | 71794 | 413 | 89.72 |
| Fucoidan 4 | 79828 | 74791 | 71610 | 416 | 89.71 |
| Fucoidan 5 | 80191 | 75254 | 74699 | 418 | 93.15 |
| Fucoidan 6 | 80404 | 75780 | 74759 | 417 | 92.98 |
| Fucoidan 7 | 80046 | 75151 | 70865 | 418 | 88.53 |
| Fucoidan 8 | 80033 | 74729 | 65760 | 420 | 82.17 |
| Dfuc1 | 79993 | 74382 | 73951 | 422 | 92.45 |
| Dfuc2 | 79462 | 74750 | 74193 | 420 | 93.37 |
| Dfuc3 | 79910 | 75113 | 73869 | 416 | 92.44 |
| Dfuc4 | 79835 | 74858 | 74177 | 419 | 92.91 |
| Dfuc5 | 79549 | 74463 | 69552 | 417 | 87.43 |
| Dfuc6 | 80117 | 75135 | 74337 | 423 | 92.79 |
| Dfuc7 | 80220 | 74753 | 71834 | 415 | 89.55 |
| Dfuc8 | 79965 | 74738 | 73986 | 424 | 92.52 |
